# Supplementary material for: Cell-type-specific plasticity of inhibitory interneurons in the rehabilitation of auditory cortex after peripheral damage
Source: Nat Commun. 2023 Jul 13;14:4170. doi: 10.1038/s41467-023-39732-7 (PMC10345144; doi:10.1038/s41467-023-39732-7)
Supplement: Supplementary file 1 — Supplementary Infomation [file 41467_2023_39732_MOESM1_ESM.pdf]

Supplementry Information

Title: Cell-type-specific plasticity of inhibitory interneurons in the rehabilitation of auditory cortex after peripheral damage

Authors: Manoj Kumar[1#], Gregory Handy[2], Stylianos Kouvaros[1], Yanjun Zhao[1], Lovisa Ljungqvist Brinson[1], Eric Wei[1], Brandon Bizup[1], Brent Doiron[2], and Thanos Tzounopoulos[1#]

[1]Pittsburgh Hearing Research Center, Department of Otolaryngology, University of Pittsburgh, Pittsburgh, PA 15261.

[2]Departments of Neurobiology and Statistics, University of Chicago

# Correspondence: Thanos@pitt.edu (TT) or Mak328@pitt.edu (MK)

Supplementary Table 1: Statistical detailed values for figure 1.

Figure	Comparison	*Statistical test*	F, *p*	N
1e	Sham-Exposed vs Noise-exposed	2-way ANOVA<br>Exposure x time interaction<br>Effect of Exposure	F =21.7, $p = 2.8 \times 10^{-12}$<br>F= 221.3, $p = 1.4 \times 10^{-34}$	Noise: 35 mice<br>Sham: 19 mice
1e	Pre NE vs NE at 1, 3, 10 days	Holm-Bonferroni's post hoc<br>Pre NE vs NE day 1<br>Pre NE vs NE day 3<br>Pre NE vs NE day 10	$p = 5.8 \times 10^{-14}$<br>$p = 1 \times 10^{-15}$<br>$p = 5 \times 10^{-9}$	35 mice
1e	Pre SE vs SE at 1, 3, 10 days	Holm-Bonferroni's post hoc<br>Pre SE vs SE day 1<br>Pre SE vs SE day 3<br>Pre SE vs SE day 10	$p > 0.99$<br>$p > 0.99$<br>$p > 0.99$	19 mice
1f	Sound-Intensity vs Time	2-way ANOVA<br>Intensity x time interaction<br>Effect of Intensity<br>Effect of Time	F =61.6, $p < 1 \times 10^{-15}$<br>F= 115.5, $p < 1 \times 10^{-15}$<br>F = 98.19, $p = 2.0 \times 10^{-14}$	35 mice
1g	Sham-Exposed vs Noise-exposed	2-way ANOVA<br>Exposure x time interaction<br>Effect of Exposure	F =15.25, $p = 9.4 \times 10^{-9}$<br>F= 56.4, $p = 7.0 \times 10^{-10}$	Noise: 35 mice<br>Sham: 19 mice
1g	Pre NE vs NE at 1, 3, 10 days	Holm-Bonferroni's post hoc<br>Pre NE vs NE day 1<br>Pre NE vs NE day 3<br>Pre NE vs NE day 10	$p = 1.9 \times 10^{-14}$<br>$p = 5.3 \times 10^{-9}$<br>$p = 2.5 \times 10^{-6}$	35 mice
1g	Pre SE vs SE at 1, 3, 10 days	Holm-Bonferroni's post hoc<br>Pre SE vs SE day 1<br>Pre SE vs SE day 3<br>Pre SE vs SE day 10	$p > 0.99$<br>$p > 0.99$<br>$p = 0.38$	19 mice
1h	Sham-Exposed vs Noise-exposed	2-way ANOVA<br>Exposure x time interaction<br>Effect of Exposure	F =8.4, $p = 5.9 \times 10^{-5}$<br>F= 11.6, $p = 1.9 \times 10^{-3}$	Noise: 20 mice<br>Sham: 10 mice
1h	Pre NE vs NE at 1, 3, 10 days	Holm-Bonferroni's post hoc<br>Pre NE vs NE day 1<br>Pre NE vs NE day 3<br>Pre NE vs NE day 10	$p = 7.2 \times 10^{-5}$<br>$p = 1.1 \times 10^{-4}$<br>$p = 4.8 \times 10^{-4}$	20 mice
1h	Pre SE vs SE at 1, 3, 10 days	Holm-Bonferroni's post hoc<br>Pre SE vs SE day 1<br>Pre SE vs SE day 3<br>Pre SE vs SE day 10	$p = 0.13$<br>$p > 0.99$<br>$p = 0.92$	10 mice
1i	Sham-Exposed vs Noise-exposed	2-way ANOVA<br>Exposure x time interaction<br>Effect of Exposure	F =13.2, $p = 3.7 \times 10^{-7}$<br>F= 21.8, $p = 6.7 \times 10^{-5}$	Noise: 20 mice<br>Sham: 10 mice
1i	Pre NE vs NE at 1, 3, 10 days	Holm-Bonferroni's post hoc<br>Pre NE vs NE day 1<br>Pre NE vs NE day 3<br>Pre NE vs NE day 10	$p = 7.3 \times 10^{-8}$<br>$p = 2.6 \times 10^{-4}$<br>$p = 1.3 \times 10^{-3}$	20 mice
1i	Pre SE vs SE at 1, 3, 10 days	Holm-Bonferroni's post hoc<br>Pre SE vs SE day 1<br>Pre SE vs SE day 3<br>Pre SE vs SE day 10	$p = 0.53$<br>$p > 0.99$<br>$p > 0.99$	10 mice
1j	Sham-Exposed vs Noise-exposed	2-way ANOVA<br>Exposure x time interaction<br>Effect of Exposure	F =16.4, $p = 1.6 \times 10^{-8}$<br>F= 8.8, $p = 6.0 \times 10^{-3}$	Noise: 20 mice<br>Sham: 10 mice
1j	Pre NE vs NE at 1, 3, 10 days	Holm-Bonferroni's post hoc<br>Pre NE vs NE day 1<br>Pre NE vs NE day 3<br>Pre NE vs NE day 10	$p = 8.3 \times 10^{-9}$<br>$p = 2.4 \times 10^{-6}$<br>$p = 9.3 \times 10^{-8}$	20 mice
1j	Pre SE vs SE at 1, 3, 10 days	Holm-Bonferroni's post hoc		10 mice

		Pre SE vs SE day 1<br>Pre SE vs SE day 3<br>Pre SE vs SE day 10	$p = 0.92$<br>$p > 0.99$<br>$p > 0.66$	
1k	Sham-Exposed vs Noise-exposed	2-way ANOVA<br>Exposure x time interaction<br>Effect of Exposure	 $F = 17.0$, $p = 2.8 \times 10^{-9}$<br>$F = 67.9$, $p = 1.0 \times 10^{-10}$	 Noise: 5 mice<br>Sham: 4 mice
1k	Pre NE vs NE at 1, 3, 10 days	Holm-Bonferroni's post hoc<br>Pre NE vs NE day 1<br>Pre NE vs NE day 3<br>Pre NE vs NE day 10	 $p = 8.0 \times 10^{-10}$<br>$p = 3.0 \times 10^{-8}$<br>$p = 5.6 \times 10^{-12}$	5 mice
1k	Pre SE vs SE at 1, 3, 10 days	Holm-Bonferroni's post hoc<br>Pre SE vs SE day 1<br>Pre SE vs SE day 3<br>Pre SE vs SE day 10	 $p = 0.07$<br>$p > 0.99$<br>$p > 0.99$	4 mice
1m	Sham-Exposed vs Noise-exposed	2-way ANOVA<br>Exposure x Frequency interaction<br>Effect of Exposure	 $F = 24.2$, $p = 1.1 \times 10^{-9}$<br>$F = 126.2$, $p = 3.6 \times 10^{-13}$	 Noise: 5 mice<br>Sham: 4 mice
1m	Sham-Exposed vs Noise-exposed	Holm-Bonferroni's post hoc<br>8 kHz<br>12 kHz<br>16 kHz<br>24 kHz<br>32 kHz	 $p > 0.99$<br>$p = 0.47$<br>$p = 0.01$<br>$p = 1.6 \times 10^{-8}$<br>$p = 6.8 \times 10^{-13}$	Noise: 5 mice<br>Sham: 4 mice

Supplementary Table 2: Statistical detailed values for figure 2.

Figure	Comparison	*Statistical test*	F, *p*	N
2d	Sham-Exposed vs Noise-exposed	Mixed model ANOVA<br>Exposure x time interaction<br>Effect of Exposure	 F =30.4, p = 4.3×10^{-9}<br>F= 63.5, p = 3.8×10^{-6}	Noise: Pre: 11 mice, d1: 8 mice, d3: 10 mice, and d10: 8 mice<br>Sham: 3 mice
2d	Pre NE vs NE at 1, 3, 10 days	Holm-Bonferroni's post hoc<br>Pre NE vs NE day 1<br>Pre NE vs NE day 3<br>Pre NE vs NE day 10	 p < 1×10^{-15}<br>p = 2.6×10^{-11}<br>p = 0.002	Pre: 11 mice, d1: 8 mice, d3: 10 mice, and d10: 8 mice 8-11 mice
2d	Pre SE vs SE at 1, 3, 10 days	Holm-Bonferroni's post hoc<br>Pre SE vs SE day 1<br>Pre SE vs SE day 3<br>Pre SE vs SE day 10	 p > 0.99<br>p > 0.99<br>p > 0.99	3 mice
2e	Sound-Intensity vs Time	Mixed-Model ANOVA<br>Intensity x time interaction<br>Effect of Intensity<br>Effect of Time	 F = 5.06, p = 1.2×10^{-13}<br>F= 53.41, p < 1×10^{-15}<br>F = 66.18, p < 1×10^{-15}	Pre: 11 mice, d1: 8 mice, d3: 10 mice, and d10: 8 mice
2e	Pre NE vs NE at 1, 3, 10 days	Holm-Bonferroni's post hoc<br>Pre NE vs NE day 1<br>Pre NE vs NE day 3<br>Pre NE vs NE day 10<br>Pre-NE vs. NE-day10 (75 and 80 dB)	 p = 2.8×10^{-5}<br>p = 0.007<br>p > 0.99<br>p < 0.04	Pre: 11 mice, d1: 8 mice, d3: 10 mice, and d10: 8 mice
2f	Sham-Exposed vs Noise-exposed	Mixed model ANOVA<br>Exposure x time interaction<br>Effect of Exposure	 F =5.1, p = 5.5×10^{-3}<br>F= 20.31, p = 7.1×10^{-4}	Noise: Pre: 11 mice, d1: 8 mice, d3: 10 mice, and d10: 8 mice 8-11 mice<br>Sham: 3 mice
2f	Pre NE vs NE at 1, 3, 10 days	Holm-Bonferroni's post hoc<br>Pre NE vs NE day 1<br>Pre NE vs NE day 3<br>Pre NE vs NE day 10	 p = 0.005<br>p = 0.001<br>p = 0.01	Pre: 11 mice, d1: 8 mice, d3: 10 mice, and d10: 8 mice
2f	Pre SE vs SE at 1, 3, 10 days	Holm-Bonferroni's post hoc<br>Pre SE vs SE day 1<br>Pre SE vs SE day 3<br>Pre SE vs SE day 10	 p > 0.99<br>p > 0.99<br>p > 0.99	3 mice
2k	Sham-Exposed vs Noise-exposed	2-way ANOVA<br>Exposure x time interaction<br>Effect of Exposure	 F =12.4, p = 5.0×10^{-8}<br>F= 11.6, p = 6.9×10^{-4}	 Noise: 358 PNs<br>Sham: 218 PNs
2k	Pre NE vs NE at 1, 3, 10 days	Holm-Bonferroni's post hoc<br>Pre NE vs NE day 1<br>Pre NE vs NE day 3<br>Pre NE vs NE day 10	 p = 2.0×10^{-10}<br>p = 4.0×10^{-4}<br>p > 0.99	358 PNs
2k	Pre SE vs SE at 1, 3, 10 days	Holm-Bonferroni's post hoc<br>Pre SE vs SE day 1<br>Pre SE vs SE day 3<br>Pre SE vs SE day 10	 p > 0.99<br>p > 0.99<br>p > 0.99	218 PNs
2l	Effect of Noise-exposure	Friedman Test<br>Pre NE vs NE day 1<br>Pre NE vs NE day 3<br>Pre NE vs NE day 10	11.95, 0.007<br>p = 0.003<br>p = 0.18<br>p > 0.99	218 PNs
2m	Sound-Intensity vs Time	2-way ANOVA<br>Intensity x time interaction<br>Effect of Intensity<br>Effect of Time	 F = 1.46, p = 0.057<br>F= 20.6, p < 1×10^{-15}<br>F = 12.5, p = 5.3×10^{-7}	218 PNs

2m	Pre NE vs NE at 1, 3, 10 days	Holm-Bonferroni's post hoc<br>Pre NE vs NE day 1<br>Pre NE vs NE day 3<br>Pre NE vs NE day 10	 $p = 0.016$<br>$p = 0.80$<br>$p = 0.001$	218 PNs
2n	Sham-Exposed vs Noise-exposed	2-way ANOVA<br>Exposure x time interaction<br>Effect of Exposure	 $F = 4.7, p = 0.002$<br>$F = 23.3, p = 1.7 \times 10^{-6}$	 Noise: 358 PNs<br>Sham: 218 PNs
2n	Pre NE vs NE at 1, 3, 10 days	Holm-Bonferroni's post hoc<br>Pre NE vs NE day 1<br>Pre NE vs NE day 3<br>Pre NE vs NE day 10	 $p = 2.0 \times 10^{-11}$<br>$p = 1.0 \times 10^{-9}$<br>$p = 2.3 \times 10^{-12}$	358 PNs
2n	Pre SE vs SE at 1, 3, 10 days	Holm-Bonferroni's post hoc<br>Pre SE vs SE day 1<br>Pre SE vs SE day 3<br>Pre SE vs SE day 10	 $p > 0.99$<br>$p > 0.13$<br>$p > 0.64$	218 PNs
2o	Effect of Noise-exposure	Friedman Test<br>Pre NE vs NE day 1<br>Pre NE vs NE day 3<br>Pre NE vs NE day 10	10.31, 0.01<br>$p = 0.003$<br>$p = 0.03$<br>$p = 0.01$	218 PNs
2q, left	Effect of Noise-exposure	1-way RM ANOVA<br>Pre NE vs NE day 1<br>Pre NE vs NE day 3<br>Pre NE vs NE day 10	$12.18, 1.0 \times 10^{-6}$<br>$p = 3.6 \times 10^{-5}$<br>$p = 0.02$<br>$p = 0.43$	175 PNs
2q, middle	Effect of Noise-exposure	1-way RM ANOVA<br>Pre NE vs NE day 1<br>Pre NE vs NE day 3<br>Pre NE vs NE day 10	$5.3, 1.0 \times 10^{-3}$<br>$p = 0.001$<br>$p = 0.012$<br>$p = 0.79$	110 PNs
2q, high	Effect of Noise-exposure	1-way RM ANOVA<br>Pre NE vs NE day 1<br>Pre NE vs NE day 3<br>Pre NE vs NE day 10	6.6, 0.0003<br>$p = 3.9 \times 10^{-5}$<br>$p = 0.001$<br>$p = 0.02$	47 PNs
2r, left	Effect of Noise-exposure	1-way RM ANOVA<br>Pre NE vs NE day 1<br>Pre NE vs NE day 3<br>Pre NE vs NE day 10	$9.6, 3.0 \times 10^{-5}$<br>$p = 6.3 \times 10^{-7}$<br>$p = 1.3 \times 10^{-5}$<br>$p = 1.1 \times 10^{-4}$	175 PNs
2r, middle	Effect of Noise-exposure	1-way RM ANOVA<br>Pre NE vs NE day 1<br>Pre NE vs NE day 3<br>Pre NE vs NE day 10	4.5, 0.01<br>$p = 2.1 \times 10^{-4}$<br>$p = 0.01$<br>$p = 0.007$	110 PNs
2r, high	Effect of Noise-exposure	1-way RM ANOVA<br>Pre NE vs NE day 1<br>Pre NE vs NE day 3<br>Pre NE vs NE day 10	4.14, 0.01<br>$p = 9.8 \times 10^{-4}$<br>$p = 1.4 \times 10^{-3}$<br>$p = 0.02$	47 PNs

Supplementary Table 3: Default parameter values.

Parameter	Value	Description
τ_m	10 (ms)	membrane time constant
τ_s	0.5 (ms)	synaptic time constant
τ_r	2 (ms)	refractory period
E_L	-65 (mV)	resting potential
V_{th}	-50 (mV)	spike threshold
V_r	-65 (mV)	reset potential
w	0.6 (mV)	synaptic strength of the excitatory connection
g	3	the synaptic factor for inhibitory connection
N_e	5000	Num. of PNs
N_p	520	Num. of PV neurons
N_s	520	Num. SOM neurons
N_v	0 / 520	Num. VIP neurons (3/4 populations)
N_{ext}^e	500	Num. external inputs to PNs
N_{ext}^p	400	Num. external inputs to PV neurons
N_{ext}^s	0	Num. external inputs to SOM neurons
N_{ext}^v	-- / 400	Num. external inputs to VIP neurons (3/4 populations)
σ_{fixed}^2	10	Fixed background noise level
r_{bg}^a	3 (Hz)	Background excitatory firing rate
r_{stim}^a	$0, 2, 4,$ or 8 (Hz)	Stimulus firing rate (none, low, med, high)
γ	0.5	Damage to background firing rate
β^a	$[0.05,0.5]$	Damage to input stimulus firing rate to pop a
I_{recov}^a	$[-5, 5]$	Recovery current to pop a
κ_{thres}	-0.7	Stability threshold

Supplement Table 4: The probability of a connection between presynaptic (columns) and postsynaptic (rows) populations.

	E	PV	SOM	VIP
E	0.03	0.10	0.10	0
PV	0.05	0.10	0.07	0
SOM	0.05	0	0	0.10
VIP	0.05	0.15	0.05	0

Supplementary Table 5: Statistical detailed values for figure 4.

Figure	Comparison	*Statistical test*	F, *p*	N
4d	Sham-Exposed vs Noise-exposed	Mixed model ANOVA<br>Exposure x time interaction<br>Effect of Exposure	 F =22.4, p = 2.8 x 10^{-7}<br>F= 89.0, p = 1.0 x 10^{-9}	Noise: Pre: 6 mice, d1: 5 mice, d3: 5 mice, and d10: 5 mice<br>Sham: 3 mice
4d	Pre NE vs NE at 1, 3, 10 days	Holm-Bonferroni's post hoc<br>Pre NE vs NE day 1<br>Pre NE vs NE day 3<br>Pre NE vs NE day 10	 p = 3.4 x 10^{-12}<br>p < 4.6 x 10^{-4}<br>p = 0.001	Pre: 6 mice, d1: 5 mice, d3: 5 mice, and d10: 5 mice
4d	Pre SE vs SE at 1, 3, 10 days	Holm-Bonferroni's post hoc<br>Pre SE vs SE day 1<br>Pre SE vs SE day 3<br>Pre SE vs SE day 10	 p > 0.99<br>p > 0.99<br>p > 0.99	3 mice
4e	Sound-Intensity vs Time	Mixed-Model ANOVA<br>Intensity x time interaction<br>Effect of Intensity<br>Effect of Time	 F = 2.9, p = 3.3 x 10^{-4}<br>F= 16.8, p < 1 x 10^{-15}<br>F = 98.5, p < 1 x 10^{-15}	Pre: 6 mice, d1: 5 mice, d3: 5 mice, and d10: 5 mice
4e	Pre NE vs NE at 1, 3, 10 days	Holm-Bonferroni's post hoc<br>Pre NE vs NE day 1<br>Pre NE vs NE day 3<br>Pre NE vs NE day 10	 p < 1.0 x 10^{-15}<br>p = 4.5 x 10^{-8}<br>p = 0.11	Pre: 6 mice, d1: 5 mice, d3: 5 mice, and d10: 5 mice
4f	Sham-Exposed vs Noise-exposed	Mixed model ANOVA<br>Exposure x time interaction<br>Effect of Exposure	 F =7.4, p = 9.6 x 10^{-4}<br>F= 53.7, p = 1.1 x 10^{-7}	Noise: Pre: 6 mice, d1: 5 mice, d3: 5 mice, and d10: 5 mice<br>Sham: 3 mice
4f	Pre NE vs NE at 1, 3, 10 days	Holm-Bonferroni's post hoc<br>Pre NE vs NE day 1<br>Pre NE vs NE day 3<br>Pre NE vs NE day 10	 p = 4.8 x 10^{-6}<br>p = 6.6 x 10^{-3}<br>p = 2.2 x 10^{-6}	Pre: 6 mice, d1: 5 mice, d3: 5 mice, and d10: 5 mice<br>5-6 mice
4f	Pre SE vs SE at 1, 3, 10 days	Holm-Bonferroni's post hoc<br>Pre SE vs SE day 1<br>Pre SE vs SE day 3<br>Pre SE vs SE day 10	 p > 0.99<br>p > 0.99<br>p > 0.99	3 mice
4k	Sham-Exposed vs Noise-exposed	2-way ANOVA<br>Exposure x time interaction<br>Effect of Exposure	 F =7.3, p = 8.5 x 10^{-5}<br>F= 11.1, p = 0.001	 Noise: 82 PVs<br>Sham: 80 PVs
4k	Pre NE vs NE at 1, 3, 10 days	Holm-Bonferroni's post hoc<br>Pre NE vs NE day 1<br>Pre NE vs NE day 3<br>Pre NE vs NE day 10	 p = 4.9 x 10^{-6}<br>p = 5.0 x 10^{-4}<br>p > 0.99	82 PVs
4k	Pre SE vs SE at 1, 3, 10 days	Holm-Bonferroni's post hoc<br>Pre SE vs SE day 1<br>Pre SE vs SE day 3<br>Pre SE vs SE day 10	 p > 0.99<br>p > 0.99<br>p > 0.99	80 PVs
4l	Effect of Noise-exposure	Friedman Test<br>Pre NE vs NE day 1<br>Pre NE vs NE day 3<br>Pre NE vs NE day 10	11.5, 0.003<br>p = 0.005<br>p = 0.003<br>p = 0.14	82 PVs
4m	Sound-Intensity vs Time	2-way ANOVA<br>Intensity x time interaction<br>Effect of Intensity<br>Effect of Time	 F = 2.2, p = 0.00034<br>F= 15.7, p < 1 x 10^{-10}<br>F = 29.8, p < 1 x 10^{-10}	82 PVs
4m	Pre NE vs NE at 1, 3, 10 days	Holm-Bonferroni's post hoc<br>Pre NE vs NE day 1<br>Pre NE vs NE day 3<br>Pre NE vs NE day 10	 p = 0.016<br>p = 0.80<br>p = 0.001	82 PVs

4n	Sham-Exposed vs Noise-exposed	2-way ANOVA<br>Exposure x time interaction<br>Effect of Exposure	 F =10.19, p = 1.6 x 10^{-6}<br>F= 26.9, p = 6.1 x 10^{-7}	 Noise: 82 PVs<br>Sham: 80 PVs
4n	Pre NE vs NE at 1, 3, 10 days	Holm-Bonferroni's post hoc<br>Pre NE vs NE day 1<br>Pre NE vs NE day 3<br>Pre NE vs NE day 10	 p = 1.6 x 10^{-9}<br>p < 1.0 x 10^{-15}<br>p = 5.2 x 10^{-7}	82 PVs
4n	Pre SE vs SE at 1, 3, 10 days	Holm-Bonferroni's post hoc<br>Pre SE vs SE day 1<br>Pre SE vs SE day 3<br>Pre SE vs SE day 10	 p > 0.99<br>p > 0.13<br>p > 0.64	80 PVs
4o	Effect of Noise-exposure	Friedman Test<br>Pre NE vs NE day 1<br>Pre NE vs NE day 3<br>Pre NE vs NE day 10	12.2, 0.002<br>p = 0.003<br>p = 0.001<br>p = 0.04	82 PVs
4q, left	Effect of Noise-exposure	1-way RM ANOVA<br>Pre NE vs NE day 1<br>Pre NE vs NE day 3<br>Pre NE vs NE day 10	12.47, 4.1 x 10^{-7}<br>p = 5.3 x 10^{-8}<br>p = 0.005<br>p = 0.17	39 PVs
4q, middle	Effect of Noise-exposure	1-way RM ANOVA<br>Pre NE vs NE day 1<br>Pre NE vs NE day 3<br>Pre NE vs NE day 10	4.3, 0.007<br>p = 0.04<br>p = 0.0009<br>p = 0.34	24 PVs
4q, high	Effect of Noise-exposure	1-way RM ANOVA<br>Pre NE vs NE day 1<br>Pre NE vs NE day 3<br>Pre NE vs NE day 10	2.8, 0.002<br>p = 0.02<br>p = 0.05<br>p = 0.72	18 PVs
4r, left	Effect of Noise-exposure	1-way RM ANOVA<br>Pre NE vs NE day 1<br>Pre NE vs NE day 3<br>Pre NE vs NE day 10	8.8, 2.0 x 10^{-5}<br>p = 1.0 x 10^{-5}<br>p = 3.5 x 10^{-5}<br>p = 6.0 x 10^{-3}	39 PVs
4r, middle	Effect of Noise-exposure	1-way RM ANOVA<br>Pre NE vs NE day 1<br>Pre NE vs NE day 3<br>Pre NE vs NE day 10	7.4, 0.0002<br>p = 0.02<br>p = 1.4 x 10^{-5}<br>p = 0.01	24 PVs
4r, high	Effect of Noise-exposure	1-way RM ANOVA<br>Pre NE vs NE day 1<br>Pre NE vs NE day 3<br>Pre NE vs NE day 10	3.06, 0.03<br>p = 0.006<br>p = 0.03<br>p = 0.006	18 PVs

Supplementary Table 6: Statistical detailed values for figure 5.

Figure	Comparison	*Statistical test*	F, *p*	N
5d	Sham-Exposed vs Noise-exposed	Mixed model ANOVA<br>Exposure x time interaction<br>Effect of Exposure	 $F = 81.34$, $p = 1.6 \times 10^{-14}$<br>$F = 682.0$, $p < 1.0 \times 10^{-15}$	Noise: Pre: 6 mice, d1: 3 mice, d3: 7 mice, and d10: 6 mice<br>Sham: 4 mice
5d	Pre NE vs NE at 1, 3, 10 days	Holm-Bonferroni's post hoc<br>Pre NE vs NE day 1<br>Pre NE vs NE day 3<br>Pre NE vs NE day 10	 $p < 1 \times 10^{-15}$<br>$p < 1 \times 10^{-15}$<br>$p < 1 \times 10^{-15}$	Pre: 6 mice, d1: 3 mice, d3: 7 mice, and d10: 6 mice
5d	Pre SE vs SE at 1, 3, 10 days	Holm-Bonferroni's post hoc<br>Pre SE vs SE day 1<br>Pre SE vs SE day 3<br>Pre SE vs SE day 10	 $p > 0.99$<br>$p > 0.99$<br>$p > 0.99$	4 mice
5e	Sound-Intensity vs Time	Mixed-Model ANOVA<br>Intensity x time interaction<br>Effect of Intensity<br>Effect of Time	 $F = 2.8$, $p = 2.3 \times 10^{-5}$<br>$F = 6.7$, $p = 4.0 \times 10^{-6}$<br>$F = 82.9$, $p < 1 \times 10^{-10}$	Pre: 6 mice, d1: 3 mice, d3: 7 mice, and d10: 6 mice<br>5-6 mice
5e	Pre NE vs NE at 1, 3, 10 days	Holm-Bonferroni's post hoc<br>Pre NE vs NE day 1<br>Pre NE vs NE day 3<br>Pre NE vs NE day 10	 $p < 1.0 \times 10^{-15}$<br>$p < 1.0 \times 10^{-15}$<br>$p < 1.0 \times 10^{-15}$	Pre: 6 mice, d1: 3 mice, d3: 7 mice, and d10: 6 mice<br>5-6 mice
5f	Sham-Exposed vs Noise-exposed	Mixed model ANOVA<br>Exposure x time interaction<br>Effect of Exposure	 $F = 2.1$, $p = 0.12$<br>$F = 0.38$, $p = 0.54$	Noise: Pre: 6 mice, d1: 3 mice, d3: 7 mice, and d10: 6 mice<br>Sham: 4 mice
5k	Sham-Exposed vs Noise-exposed	2-way ANOVA<br>Exposure x time interaction<br>Effect of Exposure	 $F = 5.3$, $p = 0.001$<br>$F = 16.6$, $p = 8.2 \times 10^{-5}$	 Noise: 82 SOMs<br>Sham: 42 SOMs
5k	Pre NE vs NE at 1, 3, 10 days	Holm-Bonferroni's post hoc<br>Pre NE vs NE day 1<br>Pre NE vs NE day 3<br>Pre NE vs NE day 10	 $p = 0.0005$<br>$p = 0.0002$<br>$p = 0.03$	82 SOMs
5k	Pre SE vs SE at 1, 3, 10 days	Holm-Bonferroni's post hoc<br>Pre SE vs SE day 1<br>Pre SE vs SE day 3<br>Pre SE vs SE day 10	 $p > 0.99$<br>$p > 0.99$<br>$p = 0.37$	42 SOMs
5l	Effect of Noise-exposure	RM on-way ANOVA<br>Pre NE vs NE day 1<br>Pre NE vs NE day 3<br>Pre NE vs NE day 10	11.0, 0.00005<br>$p = 0.005$<br>$p = 0.005$<br>$p = 0.18$	82 SOMs
5m	Sound-Intensity vs Time	2-way ANOVA<br>Intensity x time interaction<br>Effect of Intensity<br>Effect of Time	 $F = 1.6$, $p = 0.02$<br>$F = 31.04$, $p < 1 \times 10^{-15}$<br>$F = 45.9$, $p < 1 \times 10^{-15}$	82 SOMs
5m	Pre NE vs NE at 1, 3, 10 days	Holm-Bonferroni's post hoc<br>Pre NE vs NE day 1<br>Pre NE vs NE day 3<br>Pre NE vs NE day 10	 $p < 1.0 \times 10^{-15}$<br>$p < 1.0 \times 10^{-15}$<br>$p = 5.4 \times 10^{-12}$	82 SOMs
5n	Sham-Exposed vs Noise-exposed	2-way ANOVA<br>Exposure x time interaction<br>Effect of Exposure	 $F = 0.28$, $p = 0.83$<br>$F = 1.2$, $p = 0.27$	 Noise: 82 SOMs<br>Sham: 42 PVs
5o	Effect of Noise-exposure	RM one-way ANOVA	1.4, 0.24	82 SOMs
5q, left	Effect of Noise-exposure	1-way RM ANOVA<br>Pre NE vs NE day 1<br>Pre NE vs NE day 3<br>Pre NE vs NE day 10	4.2, 0.007<br>$p = 0.01$<br>$p = 0.001$<br>$p = 0.01$	31 SOMs
5q, middle	Effect of Noise-exposure	1-way RM ANOVA<br>Pre NE vs NE day 1<br>Pre NE vs NE day 3	3.36, 0.001<br>$p = 0.01$<br>$p = 0.04$	34 SOMs

		Pre NE vs NE day 10	p = 0.28	
5q, high	Effect of Noise-exposure	1-way RM ANOVA<br>Pre NE vs NE day 1<br>Pre NE vs NE day 3<br>Pre NE vs NE day 10	3.5, 0.005<br>p = 0.02<br>p = 0.03<br>p = 0.23	15 SOMs
5r, left	Effect of Noise-exposure	1-way RM ANOVA	2.3, 0.08	31 SOMs
5r, middle	Effect of Noise-exposure	1-way RM ANOVA	2.4, 0.06	34 SOMs
5r, high	Effect of Noise-exposure	1-way RM ANOVA	1.2, 0.31	15 SOMs

Supplementary Table 7: Statistical detailed values for figure 6.

**Figure**	**Comparison**	***Statistical test***	**F, *p***	**Neurons(mice)**
6d	Sham-Exposed vs Noise-exposed	Mixed model ANOVA<br>Exposure x time interaction<br>Effect of Exposure	F =2.0, p = 0.16<br>F= 0.89, p = 0.34	SEday1: 20 (3); SEday10: 20 (3)<br>NEday:1 19 (3); NEday10: 20(3)
6e	Sham-Exposed vs Noise-exposed	Mixed model ANOVA<br>Exposure x time interaction<br>Effect of Exposure	F =1.6, p = 0.20<br>F= 0.07, p = 0.78	SEday1: 20 (3); SEday10: 20 (3)<br>NEday:1 19 (3); NEday10: 20(3)
6g	Sham-Exposed vs Noise-exposed	Mixed model ANOVA<br>Exposure x time interaction<br>Effect of Exposure	F =0.09, p = 0.76<br>F= 2.6, p = 0.11	SEday1: 20 (3); SEday10: 20 (3)<br>NEday:1 19 (3); NEday10: 20(3)
6h	Sham-Exposed vs Noise-exposed	Mixed model ANOVA<br>Exposure x time interaction<br>Effect of Exposure	F =1.5, p = 0.22<br>F= 0.02, p = 0.86	SEday1: 20 (3); SEday10: 20 (3)<br>NEday:1 19 (3); NEday10: 20(3)
6m	Sham-Exposed vs Noise-exposed	Mixed model ANOVA<br>Exposure x time interaction<br>Effect of Exposure	F =3.04, p = 0.08<br>F= 0.42, p = 0.51	SEday1: 20 (3); SEday10: 20 (3)<br>NEday:1 19 (3); NEday10: 20(3)

Supplementary Table 8: Statistical detailed values for figure 8.

Figure	Comparison	*Statistical test*	F, *p*	N
8d	Sham-Exposed vs Noise-exposed	Mixed model ANOVA<br>Exposure x time interaction<br>Effect of Exposure	 F =8.8, p = 0.0003<br>F= 34.6, p = 3.3×10^{-6}	Noise: Pre: 6 mice, d1: 6 mice, d3: 6 mice, and d10: 4 mice<br>Sham: 3 mice
8d	Pre NE vs NE at 1, 3, 10 days	Holm-Bonferroni's post hoc<br>Pre NE vs NE day 1<br>Pre NE vs NE day 3<br>Pre NE vs NE day 10	 p = 6.2×10^{-8}<br>p = 2.0×10^{-4}<br>p > 0.99	Pre: 6 mice, d1: 6 mice, d3: 6 mice, and d10: 4 mice
8d	Pre SE vs SE at 1, 3, 10 days	Holm-Bonferroni's post hoc<br>Pre SE vs SE day 1<br>Pre SE vs SE day 3<br>Pre SE vs SE day 10	 p > 0.99<br>p > 0.99<br>p > 0.99	3 mice
8e	Sound-Intensity vs Time	Mixed-Model ANOVA<br>Intensity x time interaction<br>Effect of Intensity<br>Effect of Time	 F = 1.02, p = 0.44<br>F= 22.8, p < 1×10^{-15}<br>F = 29.6, p = 8.1×10^{-11}	Pre: 6 mice, d1: 6 mice, d3: 6 mice, and d10: 4 mice
8e	Pre NE vs NE at 1, 3, 10 days	Holm-Bonferroni's post hoc<br>Pre NE vs NE day 1<br>Pre NE vs NE day 3<br>Pre NE vs NE day 10	 p = 5.2×10^{-7}<br>p = 0.86<br>p = 0.0002	Pre: 6 mice, d1: 6 mice, d3: 6 mice, and d10: 4 mice
8f	Sham-Exposed vs Noise-exposed	Mixed model ANOVA<br>Exposure x time interaction<br>Effect of Exposure	 F =3.1, p = 0.04<br>F= 13.5, p = 0.007	Noise: Pre: 6 mice, d1: 6 mice, d3: 6 mice, and d10: 4 mice<br>Sham: 3 mice
8f	Pre NE vs NE at 1, 3, 10 days	Holm-Bonferroni's post hoc<br>Pre NE vs NE day 1<br>Pre NE vs NE day 3<br>Pre NE vs NE day 10	 p = 0.14<br>p = 0.02<br>p = 0.11	Pre: 6 mice, d1: 6 mice, d3: 6 mice, and d10: 4 mice
8f	Pre SE vs SE at 1, 3, 10 days	Holm-Bonferroni's post hoc<br>Pre SE vs SE day 1<br>Pre SE vs SE day 3<br>Pre SE vs SE day 10	 p > 0.99<br>p > 0.99<br>p > 0.99	3 mice
8k	Sham-Exposed vs Noise-exposed	2-way ANOVA<br>Exposure x time interaction<br>Effect of Exposure	 F =4.7, p = 0.002<br>F= 6.9, p = 0.001	 Noise: 70 VIPs<br>Sham: 60 VIPs
8k	Pre NE vs NE at 1, 3, 10 days	Holm-Bonferroni's post hoc<br>Pre NE vs NE day 1<br>Pre NE vs NE day 3<br>Pre NE vs NE day 10	 p = 0.01<br>p = 0.01<br>p = 1.0×10^{-5}	70 VIPs
8k	Pre SE vs SE at 1, 3, 10 days	Holm-Bonferroni's post hoc<br>Pre SE vs SE day 1<br>Pre SE vs SE day 3<br>Pre SE vs SE day 10	 p > 0.99<br>p > 0.99<br>p = 0.78	60 VIPs
8l	Effect of Noise-exposure	One-way RM ANOVA<br>Pre NE vs NE day 1<br>Pre NE vs NE day 3<br>Pre NE vs NE day 10	8.3, 0.001<br>p = 0.03<br>p = 0.01<br>p > 0.99	70 VIPs
8m	Sound-Intensity vs Time	2-way ANOVA<br>Intensity x time interaction<br>Effect of Intensity<br>Effect of Time	 F = 2.6, p = 0.00001<br>F= 12.1, p < 1×10^{-15}<br>F = 39.9, p < 1×10^{-15}	70 VIPs
8m	Pre NE vs NE at 1, 3, 10 days	Holm-Bonferroni's post hoc<br>Pre NE vs NE day 1<br>Pre NE vs NE day 3<br>Pre NE vs NE day 10	 p = 5.1×10^{-8}<br>p = 0.004<br>p = 1.1×10^{-5}	70 VIPs

8n	Sham-Exposed vs Noise-exposed	2-way ANOVA<br>Exposure x time interaction<br>Effect of Exposure	 F =4.72, p = 0.002<br>F= 13.7, p = 0.001	 Noise: 70 VIPs<br>Sham: 60 VIPs
8n	Pre NE vs NE at 1, 3, 10 days	Holm-Bonferroni's post hoc<br>Pre NE vs NE day 1<br>Pre NE vs NE day 3<br>Pre NE vs NE day 10	 p = 0.01<br>p =0.01<br>p = 1.0×10^{-5}	70 VIPs
8n	Pre SE vs SE at 1, 3, 10 days	Holm-Bonferroni's post hoc<br>Pre SE vs SE day 1<br>Pre SE vs SE day 3<br>Pre SE vs SE day 10	 p > 0.99<br>p > 0.99<br>p > 0.78	60 VIPs
8o	Effect of Noise-exposure	Friedman test<br>Pre NE vs NE day 1<br>Pre NE vs NE day 3<br>Pre NE vs NE day 10	9.4, 0.02<br>p = 0.56<br>p = 0.25<br>p = 0.007	70 VIPs
8q, left	Effect of Noise-exposure	1-way RM ANOVA<br>Pre NE vs NE day 1<br>Pre NE vs NE day 3<br>Pre NE vs NE day 10	4.9, 0.003<br>p = 0.007<br>p = 0.01<br>p = 0.78	33 VIPs
8q, middle	Effect of Noise-exposure	1-way RM ANOVA<br>Pre NE vs NE day 1<br>Pre NE vs NE day 3<br>Pre NE vs NE day 10	2.1, 0.01<br>p = 0.01<br>p = 0.72<br>p = 0.91	18 VIPs
8q, high	Effect of Noise-exposure	1-way RM ANOVA<br>Pre NE vs NE day 1<br>Pre NE vs NE day 3<br>Pre NE vs NE day 10	3.7, 0.002<br>p = 0.05<br>p = 0.01<br>p = 0.04	17 VIPs
8r, left	Effect of Noise-exposure	1-way RM ANOVA<br>Pre NE vs NE day 1<br>Pre NE vs NE day 3<br>Pre NE vs NE day 10	6.4, 0.009<br>p = 0.01<br>p = 0.008<br>p = 0.004	33 VIPs
8r, middle	Effect of Noise-exposure	1-way RM ANOVA<br>Pre NE vs NE day 1<br>Pre NE vs NE day 3<br>Pre NE vs NE day 10	2.8, 0.04<br>p = 0.62<br>p = 0.82<br>p = 0.01	18 VIPs
8r, high	Effect of Noise-exposure	1-way RM ANOVA<br>Pre NE vs NE day 1<br>Pre NE vs NE day 3<br>Pre NE vs NE day 10	6.1, 0.002<br>p = 0.02<br>p = 0.03<br>p = 0.001	17 VIPs

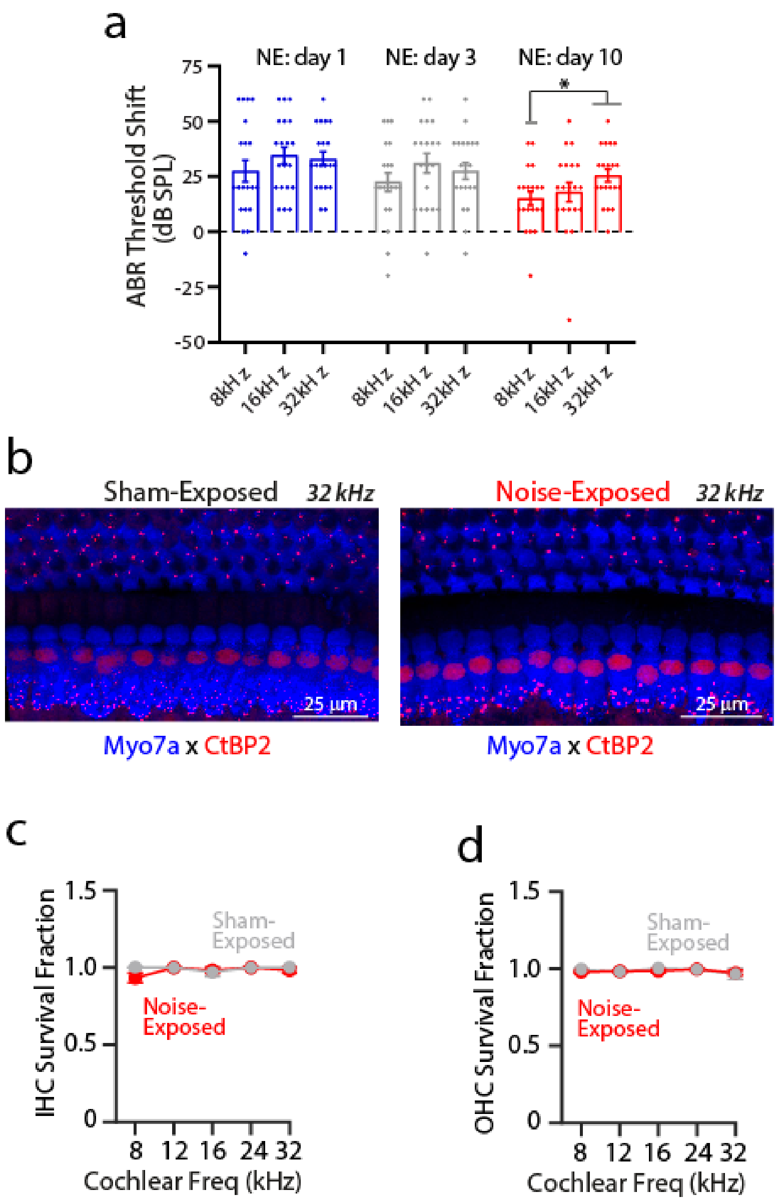

Supplementary Figure 1

Supplementary Figure 1. Noise-trauma increased the ABR thresholds for 8-32 kHz tones but did not affect either IHC or OHC survival. (a) Average ABR threshold shift to 8, 12, 16 kHz tones after NIHL. (n = 20 mice, Frequency vs. time: 2-way ANOVA; effect of frequency, F = 12.5, p = 6.4 x 10^{-5}; *, p = 2.5 x 10^{-5}, 32kHz vs 8 kHz, Holm-Bonferroni's post hoc). **(b)** Representative images of OHCs from the 32 kHz region of sham- (left) and noise- (right) exposed mice**. (c)** Quantification of IHC survival from sham- (grey) and noise- (red) exposed mice. (Noise: 5 mice vs. sham: 4 mice, 2-way ANOVA; exposure x frequency, F = 1.89, p = 0.13; effect of exposure, F = 2.7, p = 0.10). **(d)** Quantification of OHC survival from sham- (grey) and noise- (red) exposed mice. (Noise: 5 mice vs. sham: 4 mice, 2-way ANOVA; exposure x frequency, F = 0.26, p = 0.89; effect of exposure, F = 0.81, p = 0.37).

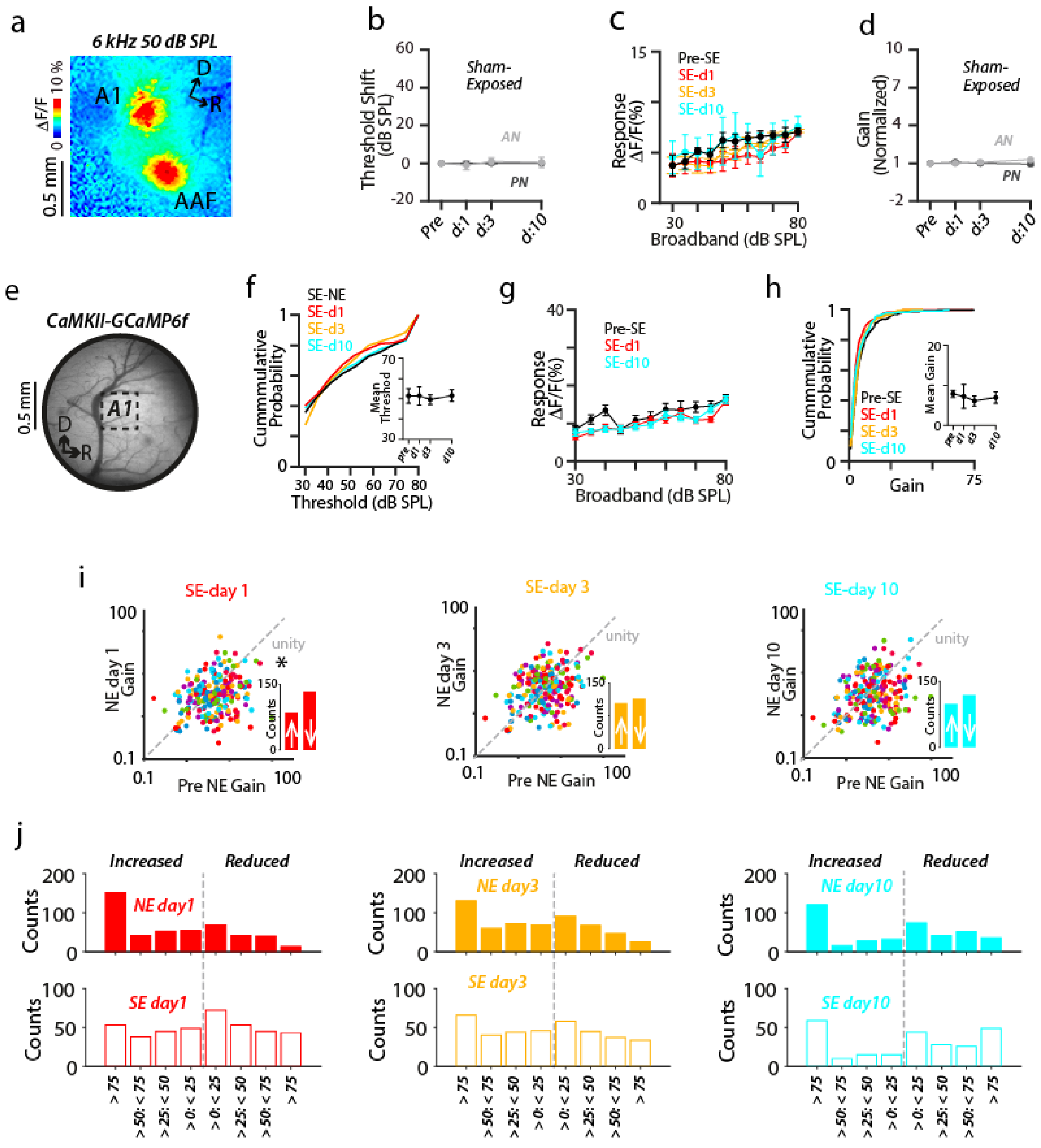

Supplementary Figure 2

Supplementary Figure 2. Sham exposure does not alter the sound-evoked activity of A1 L2/3 PNs. (a) Localization of A1. A 6 kHz 50 dB SPL tone triggered GCaMP6s fluorescence responses in two regions of the auditory cortex representing A1 and the anterior auditory field (AAF; D stands dorsal and R for rostral). **(b)** Average change in response thresholds of A1 PNs (dark grey) at 1, 3, and 10 days after sham exposure. (n = 3 mice, 1-way repeated measure ANOVA, F = 0, p > 0.99). Average change in AN threshold (light grey) reproduced from **Figure 1**. **(c)** Average responses of A1 PNs to broadband sounds from sham-exposed mice. (n = 3 mice, 2-way ANOVA; time x sound level interaction, F = 1.3, p = 0.35; effect of time, F = 0.54, p = 0.56). **(d)** Average response gain of A1 PNs (dark grey) normalized to pre-sham-exposed gain after sham exposure at 1, 3, and 10 days. (n = 3 mice, 1-way repeated measure ANOVA, F = 1.3, p = 0.34). Normalized AN gain (light grey) reproduced from **Figure 1**. **e).** Implantation of cranial glass

window over A1. **(f)** Cumulative probability of response threshold of A1 L2/3 PNs before and after sham exposure. Inset: Average mean threshold of PNs per mouse (218 PNs from 5 mice, 1-way repeated measure ANOVA, $F = 0.17$, $p = 0.87$). **(g)** Average sound-evoked responses of A1 L2/3 individual PNs to broadband sounds from sham-exposed mice. (218 PNs from 5 mice, 2-way ANOVA; sound intensity and time interaction, $F = 1.6$, $p = 0.072$; effect of time, $F = 2.9$, $p = 0.065$). **(h)** Cumulative probability of gain of A1 L2/3 PNs before and after sham exposure. Inset: Average mean gain of PNs per mouse (218 PNs from 5 mice, 1-way repeated measure ANOVA, $F = 0.29$, $p = 0.71$). **(i)** Scatter plots of the gain of individual A1 L2/3 PNs before and after sham exposure. Dotted line represents unity. Insets: Bar graphs representing the number of neurons showing increased gain (🡑 above unity) and reduced gain (🡓 below unity) after NIHL. PreSE vs. SEday1: $p = 0.006$, PreSE vs. SEday3: $p = 0.11$, and PreSE vs. SEday10: $p = 0.06$; permutation test. **(j)** Histograms showing percentage changes in the gain of L2/3 PNs after noise (top) and sham (bottom) exposure.

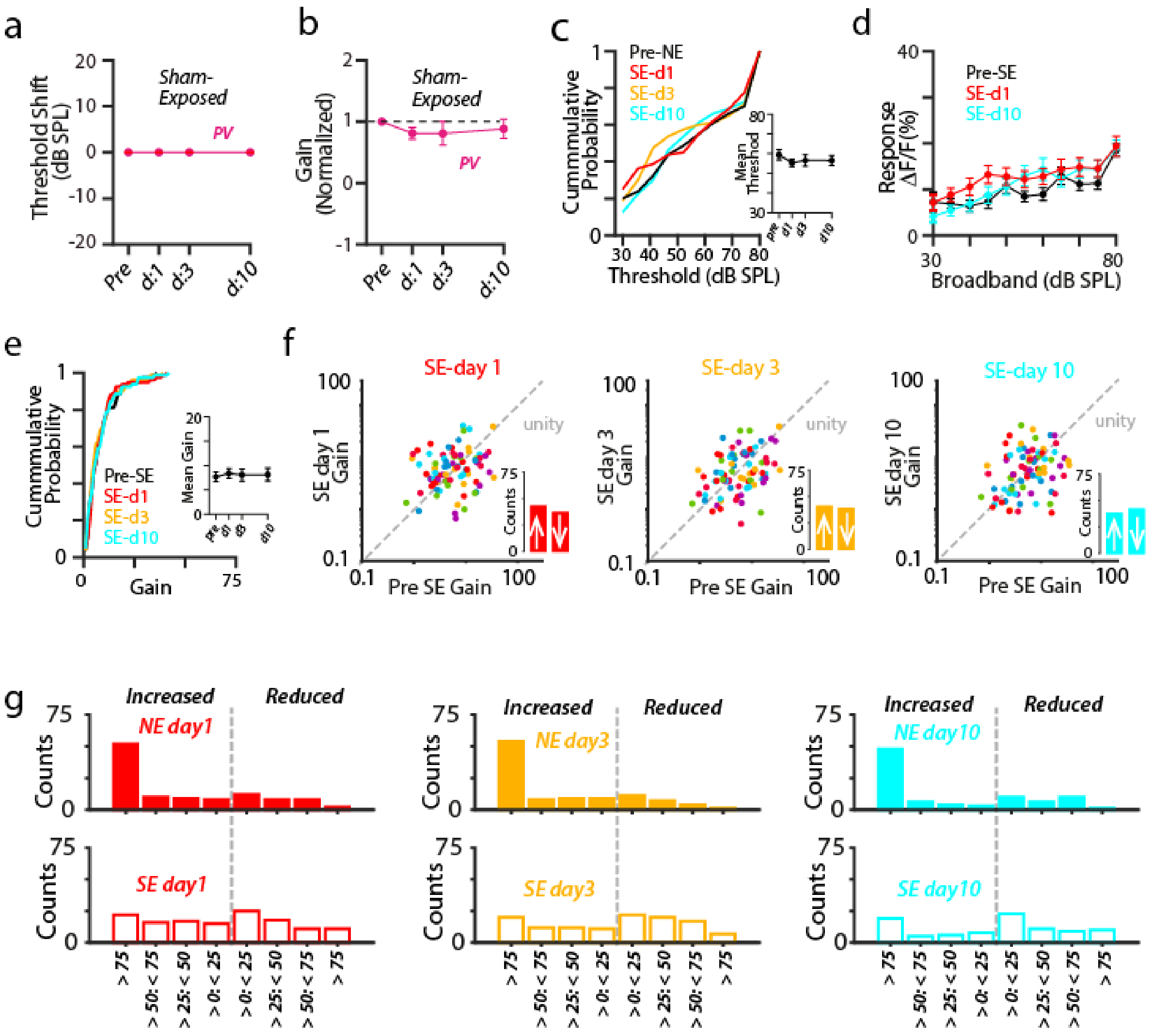

Supplementary Figure 3

Supplementary Figure 3. Sham exposure does not alter the sound-evoked activity of A1 L2/3 PVs. (a) Average change in response thresholds of A1 PVs neurons at 1, 3, and 10 days after sham exposure. (n = 3 mice, 1-way repeated measure ANOVA, F = 0, p > 0.99). **(b)** Average response gain of A1 PVs normalized to pre-sham-exposed gain after sham exposure at 1, 3, and 10 days. (n = 3 mice, 1-way repeated measure ANOVA, F = 1.09, p = 0.40). **(c)** Cumulative probability of response threshold of A1 L2/3 PV neurons before and after sham-exposure. Inset: Average mean threshold of PVs per mouse (80 neurons from 7 mice, 1-way repeated measure ANOVA, F = 0.79, p = 0.46). **(d)** Average responses of A1 L2/3 individual PVs to broadband sounds from sham-exposed mice. (80 neurons from 7 mice, 2-way ANOVA; sound intensity and time interaction, F = 1.3, p = 0.16; effect of time, F = 1.6, p = 0.16). **(e)** Cumulative gain of A1 L2/3 PVs before and after sham exposure. Inset: Average mean gain of PV neurons per mouse (80 neurons from 7 mice, 1-way repeated measure ANOVA, F = 0.14, p = 0.86). **(f)** Scatter plots of the gain of individual A1 L2/3 PVs neurons before and after sham exposure. Dotted line represents unity. Insets: Bar graphs represent the number of neurons showing increased gain (↑ above unity) and reduced gain (↓ below unity) after NIHL. PreSE vs. SEday1: p = 0.98, PreSE vs. SEday3: p = 0.67, and PreSE vs. SEday10: p = 0.96; permutation test. **g)** Histograms showing percentage changes in the gain of PVs after noise (top) and sham (bottom) exposure.

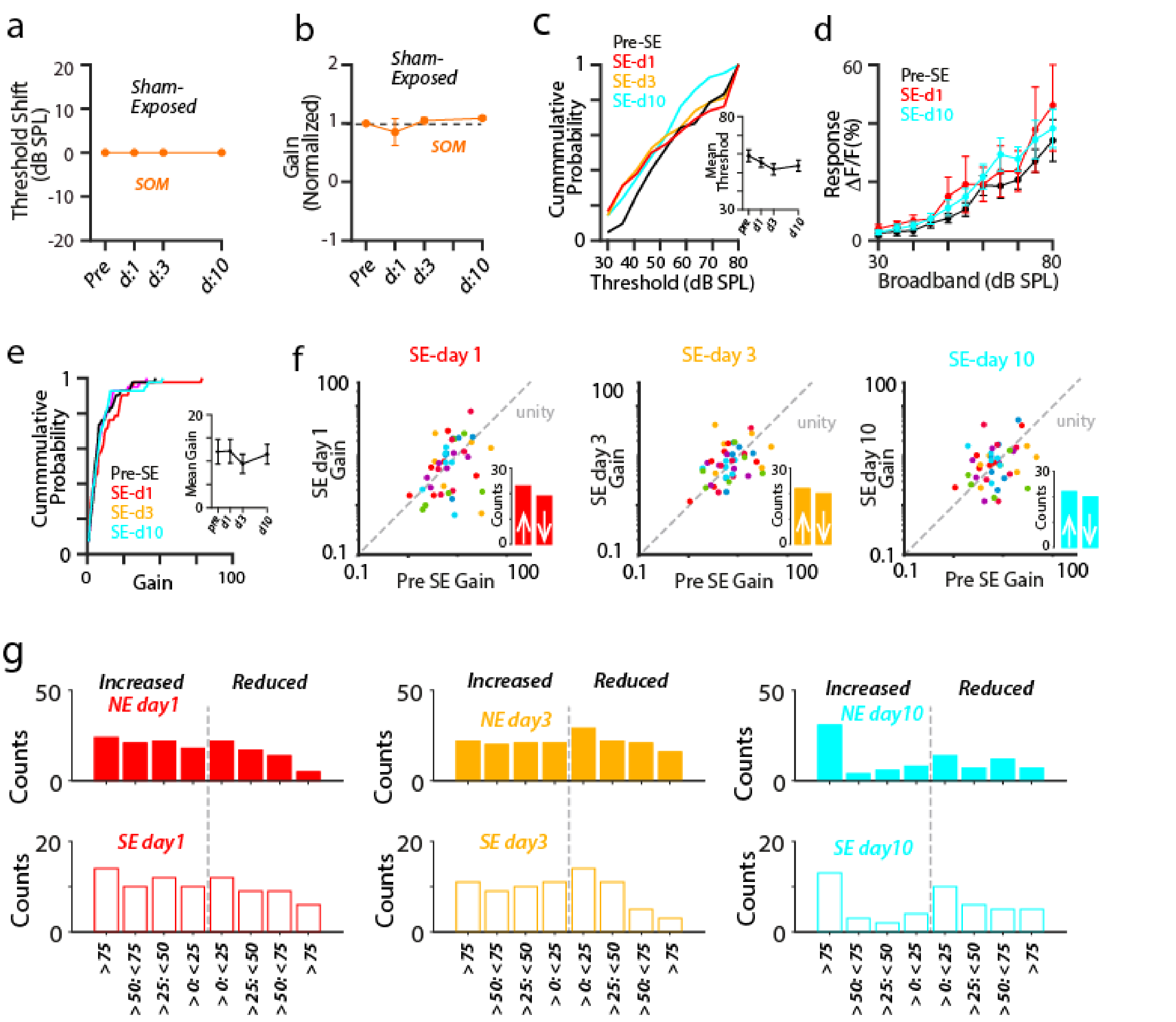

Supplementary Figure 4

Supplementary Figure 4. Sham exposure did not alter the sound-evoked activity of A1 L2/3 SOMs. (a) Average change in response thresholds of A1 SOMs at 1, 3, and 10 days after sham exposure. (n = 4 mice, 1-way repeated measure ANOVA, F = 0, p > 0.99). **(b)** Average response gain of A1 SOMs normalized to pre-sham-exposed gain after sham exposure at 1, 3, and 10 days. (n = 4 mice, 1-way repeated measure ANOVA, F = 0.85, p = 0.42). **(c)** Cumulative probability of response threshold of A1 L2/3 SOMs before and after sham exposure. Inset: Average threshold of SOMs per mouse (42 neurons from 9 mice, 1-way repeated measure ANOVA, F = 1.1, p = 0.33). **(d)** Average responses of A1 L2/3 individual SOMs to broadband sounds from sham-exposed mice. (42 neurons from 9 mice, 2-way ANOVA; sound intensity and time interaction, F = 0.92, p = 0.43; effect of time, F = 0.62, p = 0.49). **(e)** Cumulative probability of gain of A1 L2/3 SOMs neurons before and after sham exposure. Inset: Average gain of SOMs per mouse (42 neurons from 9 mice, 1-way repeated measure ANOVA, F = 0.35, p = 0.70). **(f)** Scatter plots of the gain of individual A1 L2/3 SOMs before and after sham exposure. Dotted line represents unity. Insets: Bar graphs representing the number of neurons showing increased gain (↑ above unity) and reduced gain (↓ below unity) after NIHL. PreSE vs. SEday1: p = 0.55, PreSE vs. SEday3: p = 0.94, and PreSE vs. SEday10: p = 0.89; permutation test. **(g)** Histograms showing percentage changes in the gain of L2/3 SOMs after noise (top) and sham (bottom) exposure.

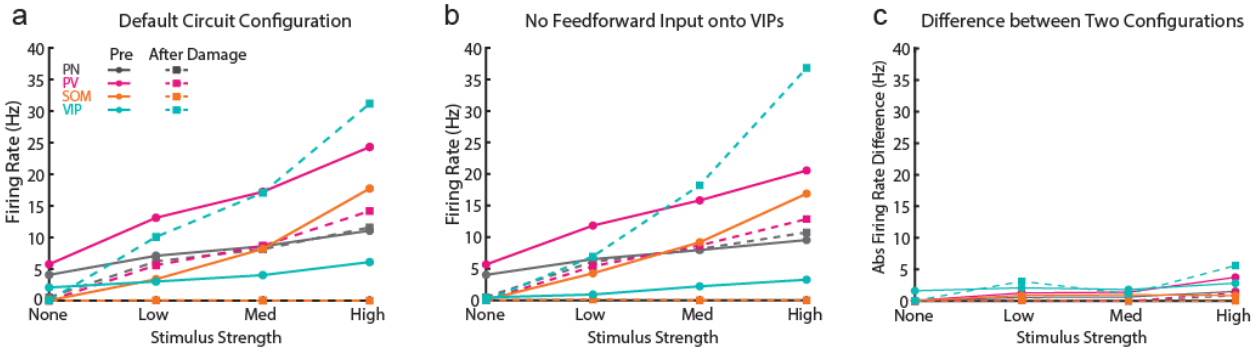

Supplementary Figure 5

Supplementary Figure 5. Direct stimulus input onto VIPs can be captured by short-term facilitation from recurrent PN connections. (a) Example viable parameter set showing all population firing rates for the pre-damage (solid lines with dots) and recovered (dashed lines with squares) states using the default network configuration illustrated in Figure 7a. **(b)** Same parameter regime as (a), except the direct stimulus input on VIPs was removed and short-term facilitation from PNs was added. Short-term facilitation was modeled by increasing the connection strength of PN→VIP (i.e., $wVIP, PN$) as a function of the PN firing rate ($w_{VIP,PN}$ varied from 0.6 to 1.3 as the stimulus strength varied from none to high). Other parameter values can be found in Tables 1 and 2. **(c)** The absolute firing rate difference between the model used in panels **(a)** and **(b)**.

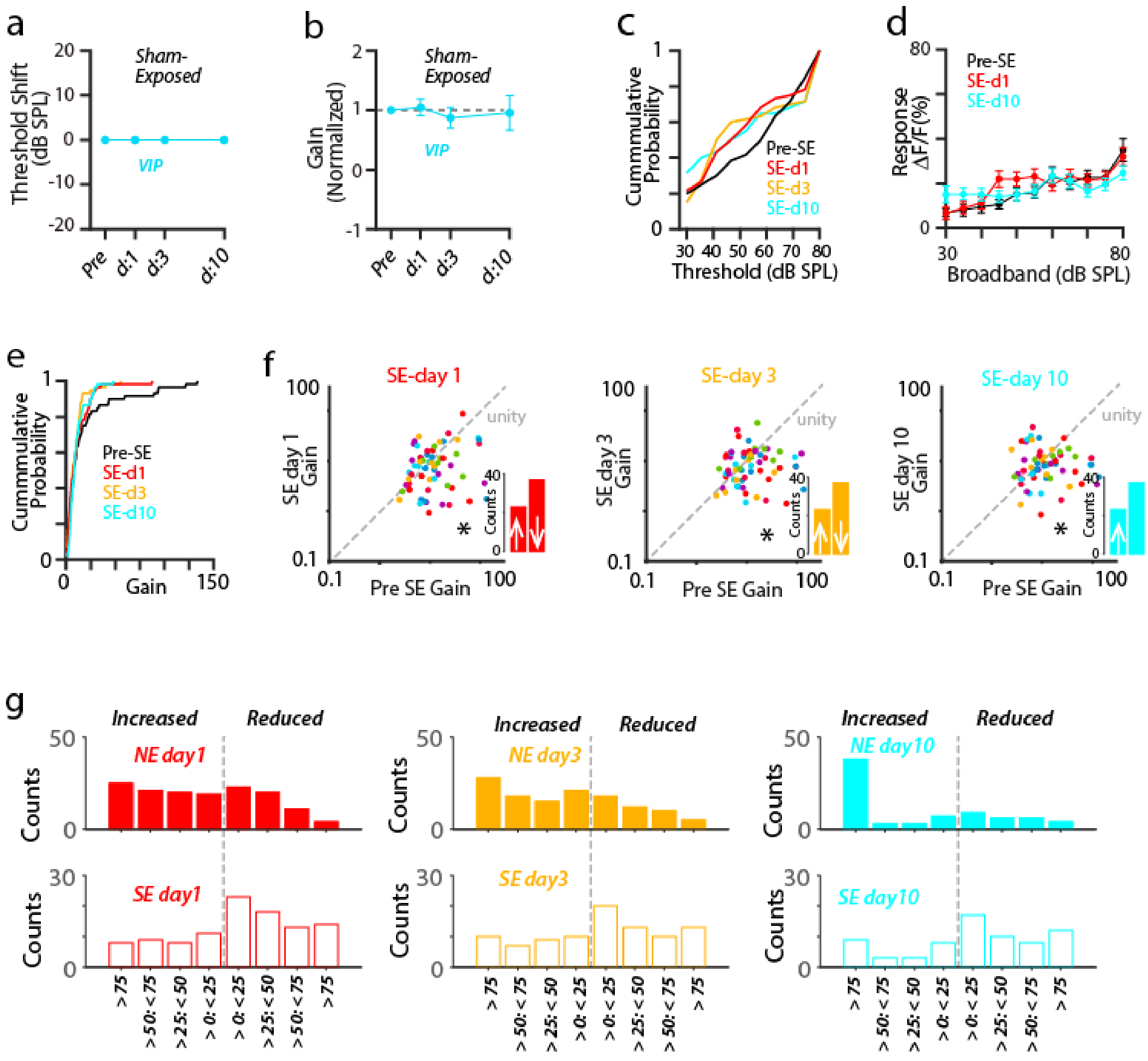

Supplementary Figure 6

Supplementary Figure 6. Sham exposure reduced the gain of A1 L2/3 VIPs. (a) Average change in response thresholds of A1 VIPs at 1, 3, and 10 days after sham exposure. (n = 3 mice, 1-way repeated measure ANOVA, F = 0, p > 0.99). **(b)** Average response gain of A1 VIPs normalized to pre-sham-exposed gain after sham exposure at 1, 3, and 10 days. (n = 3 mice, 1-way repeated measure ANOVA, F = 0.03, p = 0.98). **(c)** Cumulative probability of response threshold of A1 L2/3 VIPs before and after sham-exposure. Inset: Average mean threshold of VIP neurons per mouse (60 neurons from 6 mice, 1-way repeated measure ANOVA, F = 0.52, p = 0.62). **(d)** Average responses of A1 L2/3 individual VIPs to broadband sounds from sham-exposed mice. (60 neurons from 6 mice, 2-way ANOVA; sound intensity and time interaction, F = 4.5, p = 0.01; effect of time, F = 0.49, p = 0.69). **(e)** Cumulative probability of gain of A1 L2/3 VIPs before and after sham exposure. Inset: Average gain of VIPs per mouse (60 neurons from 6 mice , 1-way repeated measure ANOVA, F = 0.44, p = 0.61). **(f)** Scatter plots of the gain of individual A1 L2/3 VIPs before and after sham exposure. Dotted line represents unity. Insets: Bar graphs representing number of neurons showing increased gain (🡩 above unity) and reduced gain (🡫 below unity) after NIHL. PreSE vs. SEday1: p = 0.01, PreSE vs. SEday3: p = 0.005, and PreSE vs. SEday10: p = 0.006; permutation test. **(g)** Histograms showing percentage changes in the gain of L2/3 VIP neurons after noise (top) and sham (bottom) exposure.
